# Psychometric characteristics of the Center for Epidemiologic Studies Depression Scale–Revised in a population of Polish university students

**DOI:** 10.1371/journal.pone.0290536

**Published:** 2023-09-26

**Authors:** Martyna Kotyśko, Joanna Frankowiak

**Affiliations:** Department of Clinical Psychology, Development and Education, Faculty of Social Sciences, University of Warmia and Mazury in Olsztyn, Olsztyn, Poland; University of Huelva: Universidad de Huelva, SPAIN

## Abstract

The Center for Epidemiologic Studies Depression Scale–Revised (CESD-R) has already been validated on the Polish population. However, the structure of the scale obtained at that time was not confirmed by confirmatory factor analysis (CFA) in subsequent studies on the Polish sample and measurement invariance for gender was not performed. The purpose of this article is to present the results of psychometric analyses to verify the structure of the CESD-R in a university student sample. An online cross-sectional study was conducted with 1519 university students (March/April 2021). The dataset was randomly divided into three subsets to perform principal component analysis (PCA, Subset 1) and CFA for CESD-R versions with different numbers of factors and items (Subsets 2 and 3). Measurement invariance for gender was verified for the whole sample. PCA pointed to a three-factor solution that was analyzed in the subsequent CFA. Due to high correlation coefficients between factors, further steps were taken using only one factor. For CFA, 20-, 18- and 9-item versions of the CESD-R were used, but the 9-item version obtained the best model fit parameters and was used to evaluate the measurement invariance for gender, which was confirmed. The 9-item CESD-R has the best model fit compared to other versions of this scale and is gender invariant. Further research is needed to verify the criterion validity of this shortened scale.

## Introduction

It is estimated that depression occurs in about 5% of adults [1]. Young adults are particularly vulnerable to experiencing depressive symptoms [2, 3] due to the peculiarities of the developmental period of emerging adulthood, characterized by a sense of instability, self-focus and exploration of one’s own identity [4]. The need to adapt to new conditions is a challenge and an additional burden that arises for those making decision to continue education at university [5]. The years spent at university are an increased risk period for depression onset–particularly for the occurrence of first episodes [6]. In addition to anxiety symptoms, depression is a frequently diagnosed problem among university students [7]. A nationwide survey conducted in 2019 with 4500 university students found that 30% had symptoms of depression, including 6.5% with moderate to severe depression [8].

According to an annual survey by the Association for University and College Counseling Center Directors (AUCCCD), an international organization of university representatives from the United States, Canada, Europe and Asia, in 2019 nearly 90% of counseling center directors reported an increase in the number of students seeking support [9]. Along with stress and anxiety, depression is the most diagnosed problem among students [7, 9–13]. In addition, female gender is a risk factor for experiencing depressive symptoms. This tendency occurs in the general population [14–16], as well as in university students [2, 17–19], among whom the mentioned trend was also present during pandemics [20–22].

Findings from studies conducted during the COVID-19 pandemic showed that young adults are also more susceptible to experiencing depressive symptoms compared to older adults. The study, which was carried out in 2020 during lockdown on a representative sample of Polish adults, confirms that high levels of depressive symptoms were manifested primarily by 18–29-year-olds [23]. Reports from other studies indicate that undergraduate students are more afraid of COVID-19 than graduates [24]. In addition to experiencing the same anxiety and stress related to the epidemiological situation as others, and sometimes being infected themselves or facing disease or the loss of a loved one, they experienced the stress of the enforced isolation more strongly [22].

Studies on university students mental health report that there is a strong relationship between the severity of depressive symptoms and phenomena such as suicidal ideation, suicidal feelings/ actions and problematic substance use [25, 26]. The mental health of students is also related to the course of their study. According to Eisenberg et al. [27], a higher probability of dropping out of college and also obtaining a lower grade point average (GPA) are predicted by depression.

From this perspective, it is a particularly important and urgent challenge to take action against depression, with a special focus on young adults. Untreated, it can persist for a long time and interfere with daily functioning, and in severe cases can cause substance abuse or even lead to suicidal behavior. Recognizing the warning signs and making an early diagnosis is therefore crucial to treating the symptoms of depression and preventing its recurrence.

In this context, systematic screening carried out on university campuses promotes early identification of mental health problems, their nature and, consequently, better adaptation of the offer of assistance, as well as of preventive actions [2, 28]. The use of tools with proven psychometric properties can be helpful in this process and one of the suggested tools is the Center for Epidemiologic Studies Depression Scale–Revised (CESD-R).

The CESD-R is a revised version of the CESD, created by Lenore Radloff [29] and developed by William Eaton and colleagues [30]. The updated version covers the symptoms of depression included in the DSM-IV-TR [31] and the DSM-5 classification [32]. It consists of 20 statements regarding mood and behavior over the past two weeks (in the previous version of the tool, it was 7 days) and includes nine depressive symptoms: sadness/dysphoria (2, 4, 6), loss of interest/anhedonia (8, 10), appetite (1, 18), sleep (5, 11, 19), thinking/concentration (3, 20), guilt/worthlessness (9, 17), fatigue/fatigue (7, 16), movement/agitation (12, 13) and suicidal ideation (14, 15) (https://cesd-r.com). The maximum possible score on the CESD-R scale is 80 and the minimum score is 0. The original version set the cutoff point at 16. A study by Van Dam and Earleywine [33] compared two types of classification, including a cutoff point of 16 points. This solution resulted in a significant number of false positives compared to the algorithm-based classification proposed by Eaton et al. [30].

The CESD-R scale is free, publicly available and can be used by non-mental health professionals. An additional advantage is that it can be completed in no more than 4 minutes. All of this is encouraging for widespread use of the tool in studies involving representatives of various populations (primarily general populations) in different parts of the world. Currently, 15 language versions of the scale are available (https://cesd-r.com), including a Polish version developed by Koziara [34]. The authors of the revision suggest interpreting the test score, without isolating separate components [30]. However, research using this scale results in different solutions for the number of factors extracted using principal component analysis (PCA) and exploratory factor analysis (EFA) and confirmed by confirmatory factor analysis (CFA).

The factor analysis carried out in the Polish study by Koziara made it possible to distinguish three factors: cognitive-affective (14 items), physical (3 items) and self-destructive (2 items). Factor loadings did not allow item 11 to be assigned to a specific factor [34]. To our knowledge, the structure obtained in this way–a three-factor structure–has not been subjected to CFA verification on the Polish population. In a Nigerian study conducted among students [35], the solution obtained by the Polish researcher was tested, among others, and turned out to have the best fit to the data. Furthermore, a model with two factors [33] has been analyzed. A single-factor solution including all 20 items was tested using EFA by Japanese researchers [36]. In Brazil [37] and Jordan [38], a single-factor model was verified using CFA. In the aforementioned studies, a measurement invariance analysis was performed for gender, but it should be mentioned that in the Jordanian version this analysis was conducted for the 12-item version of the CESD-R [38].

Data on the measurement of depressive symptoms using the CESD-R present inconclusive results relative to the scores obtained by men and women. In a study by Kokou-Kpolou et al. [35], the authors noted that there were no differences in resolution with a single global factor, similar to a Japanese study [36]. Information presented by Koziara [34], on the other hand, indicates a higher average score among men. In contrast, a study conducted among Iraqi students identified more depressive symptoms among women [39], but data on average scores in both groups are not available.

The above information highlights an urgent need for research on the phenomenon of depression, including use of the CESD-R, among young adults as a high-risk group and provides justification for conducting our own research. The purpose of our study was to empirically verify the psychometric properties of the CESD-R. However, due to the lack of data on the Polish population in terms of confirming the structure of the tool using CFA and measurement invariance for gender, it was decided to carry out studies that would allow such analyses.

## Materials and methods

### Procedure and participants

A cross-sectional survey was conducted among 1,519 graduate and undergraduate students from one university in northern Poland. The survey was carried out between 26 March and 28 April 2021, when overall distance learning due to the COVID-19 pandemic took place. The idea of the study was to identify the need for support based on the mental state of students (taking into account the level of stress, severity of depressive symptoms and identification of functioning areas considered problematic by them), as well as to identify sources of social support. For the purposes of this article, only information on depressive symptoms is presented.

Due to the inability to conduct face-to-face research, the survey was performed online via the "Webankieta" platform. The invitation to participate was disseminated through various social media communication channels (university, departmental, student government, university psychological support center) and during the implementation of online classes by invited academics. Messages included a link to the study, redirecting students to the platform.

It was assumed that the sample of participants from each faculty should be about 10% of the total number of students in the unit. The general response rate of all university students was 9.67%, with a range of 2.86–14.0% (mean response rate: 9.86%) over the 16 faculties.

The study was approved by the Scientific Research Ethics Committee of the University of Warmia and Mazury in Olsztyn. Informed consent was obtained online, after participants were informed about the purpose of the study, anonymity, the voluntariness of participation, the ability to opt out at any time and the use of data for research purposes. Consent to participate in the study was given anonymously online by ticking the box "I agree to participate in the study".

Table 1 gives the basic characteristics of the respondents, including their division into three subsets, their gender and the year of study. In each subset, there were less men than women, and with first year students of the first cycle having the greatest representation. Also presented for each subset are the mean scores for the 20-item version of the CESD-R.

**Table 1:** Basic characteristics of the study participants.

	Total, *N* = 1519	Subset 1, *n* = 508	Subset 2, *n* = 502	Subset 3, *n* = 509
**Gender**, **n (%)**				
Women	1172 (77.2)	402 (79.1)	375 (74.7)	395 (77.6)
Men	347 (22.8)	106 (20.9)	127 (25.3)	114 (22.4)
**Year of study, n (%)**				
1st year (bachelor or long-cycle)	544 (35.8)	184 (36.2)	187 (37.3)	173 (34.0)
2nd year (bachelor or long-cycle)	299 (19.7)	88 (17.3)	98 (19.5)	113 (22.2)
3rd year (bachelor or long-cycle)	345 (22.7)	111 (21.9)	142 (28.3)	92 (18.1)
4th year of long-cycle or 1st of second cycle	200 (13.2)	75 (14.8)	57 (11.4)	68 (13.4)
5th or 6th year of long-cycle or 2nd year of second cycle	119 (7.8)	50 (9.8)	8 (1.6)	61 (12.0)
Lack of response	12 (0.8)	0 (0.0)	10 (2.0)	2 (0.4)
**CESD-R 20,** *M* (*SD*)				
Overall score	35.61 (18.59)	37.45 (18.42)	34.99 (18.89)	34.39 (18.37)

### Measures

#### The CESD-R

In this study, the CESD-R [30] with Polish validation by Koziara [34] was used. Subjects answered questions, related to determining how often in the past two weeks they had experienced the presented states, using a five-point Likert scale. Responses on the scale are as follows: (0) *not at all or less than 1 day*; (1) *1–2 days*; (2) *3–4 days*; (3) *5–7 days*; (4) *almost every day for 2 weeks*. The range of possible scores is 0–80, with a higher score indicating a higher severity of depressive symptoms. Cronbach’s alpha coefficient had the following values in the three extracted data subsets: Subset 1, α = .93; Subset 2, α = .94; Subset 3, α = .93.

#### Sociodemographic data

Information on gender, faculty and year of study was collected using a brief self-report metric.

#### Statistical analysis

The SPSS v28 statistical package was used to perform PCA, with PROMAX rotation, reliability estimation and descriptive statistics. CFA was conducted in JASP 0.16.3.0 [40] using the Diagonally Weighted Least Squares (DWLS) estimator, due to the ordinal response scale for the depression variable. The following criteria were used for CFA to determine the goodness of fit of the model to the data, based on the values of the coefficients: Comparative Fit Index (CFI > .95), Root Mean Square Error of Approximation (RMSEA < .08) and Standardized Root Mean Squared Residual (SRMR < .08) [41]. The comparison of models within the measurement invariance for gender also used the JASP program, with significance criteria for the change in the comparison range as follows: ΔCFI < −.01, ΔRMSEA ≥ .015 and ΔSRMR ≥ .03 [42].

The dataset was randomly divided into three subsets so that all the assumed analyses could be carried out. Activities in each set were as follows: Subset 1, PCA; Subset 2, CFA for the solution obtained under PCA and for the one-factor solution; Subset 3, CFA for the one-factor solution with a reduced number of items. Entire dataset was used to obtain model fit indices of the final solution and to verify measurement invariance across gender.

## Results

### Step 1: PCA

To analyze the structure of the 20-item CESD-R, PCA was performed on Subset 1 using a non-orthogonal PROMAX rotation. The result was the extraction of four factors, which together explained about 64.5% of the variance. Analysis of the item distribution showed that one of the factors is formed inclusively of two items and there are high cross-loadings. Given that the Polish study by Koziara [34] extracted a solution for three factors, it was decided to perform PCA again but with three factors imposed. This solution explains 59.1% of the variance. A content analysis of the items included in the individual factors recognized the regularities linking the items, which resulted in the following working labels being applied: (1) Appetite & Sleep (items 1, 5, 18 and 19), (2) Suicidal Aspects (items 9, 14, 15, 17) and (3) General (items 2, 3, 4, 6, 7, 8, 10, 11, 12, 13, 16, 20). Item 11, which in content and in relation to the depression criterion is about sleep problems, was not included in the Appetite & Sleep factor. An analysis with an imposed one-factor solution was also performed, since the scale is not intended to split into factors. The percentage of explained variance is 44.6%. Two items received factor loadings lower than .4: item 11 (.363) and item 18 (.286).

### Step 2: CFA for solutions obtained under PCA and for a one-factor solution with different numbers of items

CFA was carried out on Subset 2 for the three-factor solution obtained by PCA from Step 1 and for the one-factor solution for all 20 items of the CESD-R, as well as after removing items 11 and 18. The possibility of a shortened version of the scale (9-item version) was also tested. The results are shown in Table 2.

**Table 2:** Confirmatory factor analysis results for different model solutions of the CESD-R.

Model	*χ* ^*2*^	df	*p*	CFI	RMSEA [90% CI]	SRMR
**SUBSET 2**						
3 factors (20 items)	499.77	167	< .001	.995	.063 [.057, .069]	.057
3 factors (item 11 excluded)	476.36	149	< .001	.995	.066 [.06, .073]	.058
3 factors (items 11 and 18 excluded)	416.80	132	< .001	.995	.066 [.059, .073]	.052
1 factor (20 items)	1094.24	170	< .001	.986	.104 [.098, .11]	.079
1 factor (items 11 and 18 excluded)	954.67	135	< .001	.987	.11 [.104, .117]	.076
1 factor with modifications^a^ (item 11 and 18 excluded)	326.06	132	< .001	.997	.054 [.047, .062]	.046
1 factor (with only 9 items)	40.02	27	.051	.999	.031 [.00, .05]	.037
**SUBSET 3**						
1 factor with modifications^a^ (18 items)	489.26	132	< .001	.994	.073 [.066, .08]	.053
1 factor with only 9 items	41.72	27	.035	.998	.033 [.009, .051]	.039

Note:

^a^ Modifications of the model included correlating the residuals at the following item pairs: 5–19, 14–15 and 9–17

The model assuming the existence of three factors has satisfactory parameters in each of the included solutions. When all 20 items are considered, items 11 and 18 perform the weakest compared to the others. After removing them, the model continues to show a good fit to the data but the main concern is the high correlation between the extracted factors: Appetite & Sleep and Suicidal Aspects (*r* = .54), Appetite & Sleep and General (*r* = .71) and, most importantly, Suicidal Aspects and General (*r* = .82). As Rababah et al. [38] suggest, this is a premise that contradicts the existence of independent factors. Due to this fact, measures including the three-factor model were not pursued, although a different stance was taken by Kokou-Kpolou et al. [35]. We decided to rely on the one-factor solution, which, with all items retained, initially had the weakest model fit parameters. Several modifications were made, the first being to exclude items 11 and 18 from the model because their factor loadings were the lowest, but this did not significantly improve the model parameters. The next action was to correlate the residuals for the following item pairs: 5/19, 14/15 and 9/17. This resulted in a significant improvement in the parameters of the model fit, the values of which are within the limits adopted earlier.

Taking into account the fact that modifications could be made to the CESD-R (changing the number of items and correlating the residuals), it was decided to test a radically different solution: with one factor and nine items. This was inspired by the study of Haroz, Ybarra and Eaton [43], who tested among adolescents an abbreviated version of the CESD-R scale with 10 items (nine questions taken directly from the CESD-R and one reformulated question adapted to the specifics of depression among adolescents). The use of only the 9-item version of the CESD-R allows each criterion of depression to be addressed (one item per criterion). In the Subset 2 sample, the properties of the model met the criteria for a well-fitting model and, compared to the 18-item version, performed better.

### Step 3: CFA for one-factor solution with 18- and 9-item versions of the CESD-R

Due to the promising parameters of the univariate model with the 18- and 9-item versions of the CESD-R, it was decided to test them again in Subset 3 (Table 2). The properties of the model for the 18-item version were worse than in Subset 2, whereas for the 9-item version the parameters were at a very similar level to the previous one. Table 3 presents the list of nine items included in the short version of the CESD-R, factor loadings from CFA performed on the whole sample and model fit indices.

**Table 3:** The nine item version of CESD-R, *n* = 1519.<

Item number from the original version of CESD-R 20	Item phrasing	Factor loadings
1	PL: Miałem(am) kiepski apetyt.	.467
	ENG: My appetite was poor.	
5	PL: Mój sen był niespokojny.	.578
	ENG: My sleep was restless.	
6	PL: Czułem(am) się smutny(a)	.766
	ENG: I felt sad.	
9	PL: Czułem(am) się złym człowiekiem.	.619
	ENG: I felt like a bad person.	
10	PL: Straciłem(am) zainteresowanie codziennymi zajęciami	.787
	ENG: I lost interest in my usual activities.	
12	PL: Czułem(am) jakbym poruszał(a) się zbyt wolno.	.613
	ENG: I felt like I was moving too slowly.	
14	PL: Chciałem(am) umrzeć.	.553
	ENG: I wished I were dead.	
16	PL: Cały czas byłem(am) zmęczony(a).	.744
	ENG: I was tired all the time.	
20	PL: Nie mogłem(am) skupić się na ważnych rzeczach.	.770
	ENG: I could not focus on the important things.	
Model fit indices: χ^2^ *=* 94.98, df = 27, *p* < .001; CFI = .994; RMSEA [90% CI] = .041 [.032-.050]; SRMR = .038		

Note: Polish and English versions of the items taken from: https://cesd-r.com/download/

The decision was made to check the measurement invariance for gender for the short version only. In addition, the mean results of women and men were analyzed according to the particular version of the CESD-R (Table 4). Regardless of the number of items, women scored higher each time compared to men. This is an additional justification for the implementation of measurement equivalence analysis so that the differences obtained can be properly interpreted.

**Table 4:** Comparison of men and women results for different versions of the scale.

	Women, *n* = 1172		Men, *n* = 347				
	*M*	*SD*	*M*	*SD*	*t*	*p*	*d*
CESDR-20	37.03	18.23	30.81	19.02	5.53	< .001	.34
CESDR-18	35.16	17.37	29.23	18.24	5.53	< .001	.34
CESDR-9	16.70	8.81	14.11	9.19	4.76	< .001	.29

### Step 4: Measurement invariance across gender

The measurement invariance across gender was verified on four levels (Table 5): configural, metric, scalar and strict. Configural invariance determines whether the tool has the same structure in both groups. All parameters of the presented model are satisfied. Metric invariance is referred to as weak invariance, which verifies that the path loadings take similar values in both groups. Relative to the 9-item version of the CESD-R, this form of invariance was also confirmed: the model parameters changed slightly but were within the a priori accepted limits. The more stringent scalar invariance had even better model fit parameters to the data compared to the metric level. Thus, it can be concluded that the intercepts are the same in both groups. The highest level of invariance, referred to as “strict”, indicated identical model parameters to the scalar level. Thus, it can be concluded that relative to the 9-item version of the CESD-R, there is full measurement invariance across gender.

**Table 5:** Measurement invariance of the 9-item CESD-R across gender (*n* = 1519).

Model	*χ* ^*2*^	df	CFI	RMSEA	SRMR	Δχ^2^	ΔCFI	ΔRMSEA	ΔSRMR
Configural	178.328	54	.995	.055	.046	-	-	-	-
Metric	201.089	62	.994	.054	.049	22.761	.001	-.001	.003
Scalar	232.957	88	.994	.047	.047	31.868	.000	-.007	-.002
Strict	232.957	88	.994	.047	.047	0	.000	.000	.000

Note: *P*-value for each *χ*^*2*^ test was < .001

## Discussion

This study tested the psychometric properties of the Polish version of the CESD-R among students. Based on the results presented in other studies, both three- and one-factor solutions, as well as versions with a reduced number of items, were verified. Based on the analyses, a single-factor solution with nine questions was adopted, which referred to all the criteria for depression presented in the DSM-5 classification. This version was used to verify the measurement invariance within gender and the result of this analysis confirmed that it was present at all levels.

The CESD-R, an update of a tool that has been in use for years in depression research in the general population, has been successively revised in various countries, as evidenced by its involvement in a growing number of studies. The authors of the original version [30] did not presume to extract factors but analyses conducted on the basis of the collected research data indicate that it is possible; however, in some cases the exploratory extracted multi-factor structure is confirmed, as in Kokou-Kpolou et al. [35] and in others it is not e.g., the four-factor solution from the study of Rababah et al. [38]. In a study of the Polish version of the scale, Koziara [34] extracted a three-factor solution based on EFA but without confirmation for this solution within CFA. A research team conducting a study among Nigerian students [35] using only CFA showed that the three-factor solution obtained by Koziara [34] had the best fit to the data and that in the gender invariance analysis full invariance was obtained at the configural and metric levels but only partial confirmation at the scalar and strict levels. In our own study, three factors also emerged and the model itself had good parameters; however, due to the very high intercorrelations of the factors it was decided not to subject this version to further analysis, including measurement invariance, in favour of verifying the univariate solution, which is also supported by other studies [37, 38].

Analyzing in detail the data for the items that make up the CESD-R, we observed that item 11 ("I slept much more than usual") and item 18 ("I lost a lot of weight without trying to") obtained the lowest factor loadings each time. In the Polish study by Koziara [34], item 11 was also problematic because the factor loadings did not allow it to be specifically assigned to any of the three extracted factors. Furthermore, in the Faro et al. [37] study, item 11 had the lowest factor loadings in the EFA procedure and one of the lowest loadings in the CFA. Item 18 on weight loss has not performed unequivocally poorly in other studies. In Koziara [34], item 18 had a very high factor loading in EFA (λ = .86), being part of the "physical" factor including three items. In Faro et al. [37], item 18 had a factor loading of λ = .50 in EFA and λ = .40 in CFA, both of which can be considered relatively low values. In the Rababah et al. [38] study, item 18 remained in the CESD-R after being shortened but the CFA factor loading value was the second lowest. Furthermore, in a publication by Van Dam and Earleywine [33], when testing a single-factor solution, items 11 and 18 received the lowest factor loadings of all items: .62 and .61, respectively.

The presented regularities of the indicated items are worth interpreting, taking into account the period of implementation of our own research, which to some extent can explain their low factor loadings. The research took place during the period of lockdown and distance learning at universities in Poland. This involved students staying at home or in residential facilities, without the possibility of moving to university buildings. These circumstances may have influenced the widespread increase in time spent sleeping, resulting from not having to get up early and prepare to spend the day away from home. For this reason, the increase in sleep time may not have been considered as a different condition than before. Decrease in physical activity due to restrictions caused by the COVID-19 pandemic [44] and dietary choices and eating habits [45] might have promoted weight gain. The CESD-R item on weight loss (item 18) achieved the lowest mean score (*M* = .41) in the entire sample analyzed, even lower than item 15 on the desire to harm oneself (*M* = .45). It seems, therefore, that items 11 and 18 may have worked differently in the pandemic context than in other social circumstances, although, as indicated earlier, these items, or more precisely the values of their factor loadings, are outliers in other studies.

In our own study, invariance of reduced 9-item version of CESD-R was confirmed at four levels of measurement, indicating that the results obtained with this scale can be compared across genders. Other studies using the scale, whether for the full [35] or the shortened version [38], have also considered and demonstrated gender measurement invariance analysis. Our results for the abbreviated version of the scale therefore do not differ from data collected in other countries or on age-matched and socially similar populations.

### Limitations

The present study is not free of limitations. Although it was possible to survey more than 1500 students at a Polish university, it is advisable to repeat the study outside the context of a pandemic and distance learning, which may not have been without influence on the results obtained. In the analyzed sample there was a definite predominance of women, so it would be important to ensure that the proportion of men relative to women is more balanced in future studies. Since all the participants of the study were university students, generalizing the results is limited. For this reason, it is desirable to conduct a population-based study through which not only the mental condition of students but also other age-matched and socially similar groups will be included.

## Conclusions and future directions

As presented in the Discussion, the CESD-R is successively undergoing analyses aimed at determining its basic psychometric properties and verifying its structure. Studies conducted in different countries and among different age and social groups confirm the single-factor structure of the tool (e.g. Faro et al. [37]) and also indicate the possibility of extracting more factors [34, 35] or reducing the number of items forming the scale (e.g. Rababah et al. [38]). In our presented study conducted among students during the lockdown period associated with the COVID-19 pandemic and the time of remote learning, we verified various solutions present in the literature for the CESD-R, starting with a three-factor solution and ending with a single-factor version using a reduced number of items (nine items). The selected solution was subjected to a gender-invariance analysis and this measurement invariance was confirmed, allowing mutual comparisons between the men and women tested with this version of the scale.

An important next step, taking into account the shortened 9-item version of the CESD-R, would be to verify the criterion validity of the tool. It would be desirable to test convergent validity (using other existing tools to measure depression), divergent validity (using tools to measure other variables that should not correlate with depression). A valuable action in relation to the usefulness of the shortened version of the scale would also be to collaborate with a psychiatrist who, in a follow-up, could make the diagnosis of depressive disorder. By treating, for instance, the diagnosis made by the psychiatrist as the gold standard, it would also be possible to check the sensitivity and specificity of the scale, as well as to determine, using the Receiver Operating Characteristic (ROC) curve, what number of points obtained by the person completing the tool represents the best cut-off point to be used as an indication for an individual to consult a health professional. Using a tool with proven psychometric properties can facilitate the organization of surveys among students, through which the need among them for support will be recognized. This is particularly important in view of the increasing incidence of depression in this group. In Poland, psychological support is not an obligatory part of the functioning of higher education institutions, although many universities are making changes in this regard [46]. The results of the research described in this paper were used to adjust the assistance offered in order to meet the demand of students in the target university.
